# Density-dependent predatory impacts of an invasive beetle across a subantarctic archipelago

**DOI:** 10.1038/s41598-023-41089-2

**Published:** 2023-09-02

**Authors:** Charly Géron, Ross N. Cuthbert, Hoël Hotte, David Renault

**Affiliations:** 1 University of Rennes, CNRS, ECOBIO (Écosystèmes, Biodiversité, Évolution) - UMR 6553, 263 Avenue du Général Leclerc, 35042 Rennes, France; 2 Institute for Global Food Security, School of Biological Sciences, Queen’s University Belfast, 19, Chlorine Gardens, BT9 5DL Belfast, United Kingdom; 3 Nematology Unit, Plant Health Laboratory, ANSES, Domaine de la Motte au Vicomte - BP 35327, 35650 Le Rheu, France

**Keywords:** Climate-change ecology, Invasive species, Population dynamics, Biodiversity, Ecology

## Abstract

Biological invasions represent a major threat to biodiversity, especially in cold insular environments characterized by high levels of endemism and low species diversity which are heavily impacted by global warming. Terrestrial invertebrates are very responsive to environmental changes, and native terrestrial invertebrates from cold islands tend to be naive to novel predators. Therefore, understanding the relationships between predators and prey in the context of global changes is essential for the management of these areas, particularly in the case of non-native predators. *Merizodus soledadinus* (Guérin-Méneville, 1830) is an invasive non-native insect species present on two subantarctic archipelagos, where it has extensive distribution and increasing impacts. While the biology of *M. soledadinus* has recently received attention, its trophic interactions have been less examined. We investigated how characteristics of *M. soledadinus*, its density, as well as prey density influence its predation rate on the Kerguelen Islands where the temporal evolution of its geographic distribution is precisely known. Our results show that *M. soledadinus* can have high ecological impacts on insect communities when present in high densities regardless of its residence time, consistent with the observed decline of the native fauna of the Kerguelen Islands in other studies. Special attention should be paid to limiting factors enhancing its dispersal and improving biosecurity for invasive insect species.

## Introduction

Invasive non-native species—those species that are introduced by humans, establish, spread in environments where they were not formerly found—represent a major threat to native biodiversity worldwide^1–4^. The invasion-induced threat is worsening with intensification of trade and development of new transport pathways, in addition to global warming, altogether facilitating non-native species to arrive, establish and spread in more diverse locations^1,5,6^.

The impacts caused by non-native species may be particularly pronounced towards the poles, due both to the ongoing potential for biological invasion as new trade routes open, and to climate warming^7–9^. Consistently, “polar amplification” refers to the fact that cold regions (alpine, Arctic, subarctic, Antarctic and subantarctic areas) experience faster and more intense effects of climate change, manifesting in the reduction of frozen surfaces, extreme winter warming events, precipitation regime changes, summer droughts and heat periods, among others^10,11^. These strong changes in local climatic conditions alter physiological responses of many organisms, in particular plants and ectotherms. Some native plants and insects seem to benefit from warmer conditions, but those species might suffer from increased mortality and reduced development once developmental thresholds in environmental conditions are reached, compromising ecosystem functioning^12–14^.

In a warmer world, insular communities from cold regions are also increasingly exposed to incursions of non-native species, especially when native communities are weakened due to global changes^15,16^. These native communities are often species poor, and characterized by a high degree of endemism, making non-native species a high risk to biodiversity conservation in the absence of trophically-analogous species^17^. Insular cold areas also have lower functional diversity, hence displaying vacant niches that can be readily exploited by non-native species^18, 19^, and in some cases even lack predatory species^17^. Consequently, native communities from cold regions are naive to predators, as evidenced, for example, by the fact that many native insect species are flightless; an obvious disadvantage in the face of non-native predators^20–23^.

Subantarctic islands are cold environments situated between 45 °S and 54 °S, and encompass most of the terrestrial biotas at mid and high latitudes of these locations^24^. They exhibit key characteristics—harsh conditions and geographic isolation, high sensitivity to climate change, low species diversity, limited anthropogenic influence as compared with temperate regions, and recent introductions of non-native species—making them ideal open-air laboratories to study non-native species and their impacts, many of which are relatively unknown^24,25^. Subantarctic islands have long been considered as relatively pristine areas in terms of non-native species, but the number of non-native species began increasing in the nineteenth century when sealing and whaling industries established, and the introduction of non-native species has dramatically risen in the last 60 years after the establishment of permanent research stations^26,27^. As insects are particularly responsive to environmental conditions, including climate change^28^, the impacts caused by non-native insect species establishment in these cold regions may be bolstered under warmer conditions, due to an increase in metabolism, fecundity, spread and survival^16,29,30^.

Along with competition for food and/or space, predation is a major source of demographic density dependence which greatly influences prey community structure. Predator responses to prey density can vary among species and environments, but prey consumption rates most often decrease at high prey densities, while in these conditions, insect predators often kill more prey than they eat^31,32^. Predator sex and body mass, as well as environmental conditions such as temperature increasing physiological activities, can also affect prey consumption^33,34^. For example, when prey are scarce, a greater mobility of their predators has usually been reported, which makes them more visible and increases their own predation risk if higher predators are present^35^. Moreover, effects of body size on predation can differ between sexes^36^, with interactions also mediated by sex-specific behaviors such as mate searching^37,38^. Interaction strengths towards prey additionally often peak at intermediate predator–prey body size ratios, with predators less efficient towards prey which are too large or too small^35,39,40^. Understanding the relationships between predators and prey is therefore essential as they establish the links between trophic levels, and in consequence affect species community dynamics^41–44^.

On Kerguelen Islands, mean annual air temperature has increased by 1.3 °C since the 1960s, and summer temperature extremes have been recorded, reaching up to 23 °C^11^. Non-native insect species have been introduced to the Kerguelen Islands, which now host more non-native than native arthropod species (which is also the case for other species groups, such as vascular plants, and on other subantarctic islands)^11,27^. One of those non-native insect species, the predatory carabid beetle *Merizodus soledadinus* (Guérin-Méneville, 1830) (Coleoptera: Trechidae), was accidentally introduced from the Falkland Islands in 1913 at a single site on Kerguelen Islands (Port Couvreux: 49°17′04.9″S, 69°41′41″E), where it was first identified in 1939^45^. Its distribution and density increased rapidly in the early 1990s^11,46^. Nowadays, it occupies a large range, mainly constituted of coastal environments but is also expanding along hydrographic networks, where trophic resources and water are available^47^. In its invaded ranges, *M. soledadinus* predation heavily impacts native arthropods^48^.

While the biology of *M. soledadinus* has recently received attention, its ecological impacts, in particular the trophic interactions of the species, have been less examined. The continuous monitoring of the geographic expansion of the species on Kerguelen Islands makes it possible to record the time since the arrival of *M. soledadinus* at a specific location (hereafter referred to as “residence time”), and thus indicates the spatial sorting of the populations and along the invasion fronts. This is particularly crucial information, as it has been found that individuals from the invasion front are larger^25^, and hence, may be characterized by different predation behavior^49–51^. Here, we used the detailed knowledge about the invasion process of *M. soledadinus* on Kerguelen Islands, to investigate what impact the population density of such a non-native predatory insect has on its predation rate. The following main characteristics were also considered: mean body mass, residence time, sex ratio, the number of prey, and the duration of the experiment, which allowed observations of the prey-predator interactions, i.e. the proportion of eaten, attacked and dead prey at various observation time points during the experiment. We hypothesized that the number of predators would be positively correlated with predation rates, while an opposite pattern would occur with number of prey. Additionally, we hypothesized that the residence time would affect predation, with greater predation expected by populations with a shorter residence time owing to greater boldness at the invasion fronts^49^. Finally, we derived a measure of the potential population-level impact of *M. soledadinus*, taking into account its maximum feeding rate and its abundances (based on Dick et al. 2017^52^), hypothesizing that longstanding populations will have a greater abundance, but that this will be offset by a lower *per capita* effect.

## Results

**Figure 1:**
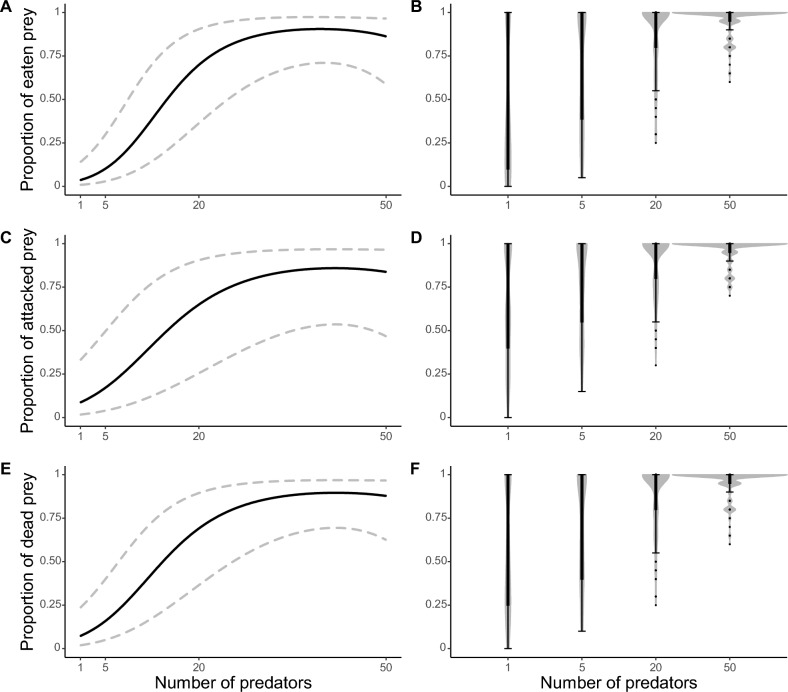
Trends for the best generalized linear mixed models testing for the drivers of the proportion of eaten, attacked and dead prey as a function of the number of predators (**A**, **C**, **E**, respectively). Lower and upper 95 % confidence intervals are represented by the dashed lines. Violin boxplots for the proportion of eaten, attacked or dead prey are a combination of kernel density plots and boxplots. For each predator density (n = 180 replicates), violin boxplots represent the distribution of the raw data, and the upper adjacent value, third quartile, median, first quartile and lower adjacent value from top to bottom (**B**,**D**,**F**, respectively).

**Figure 2:**
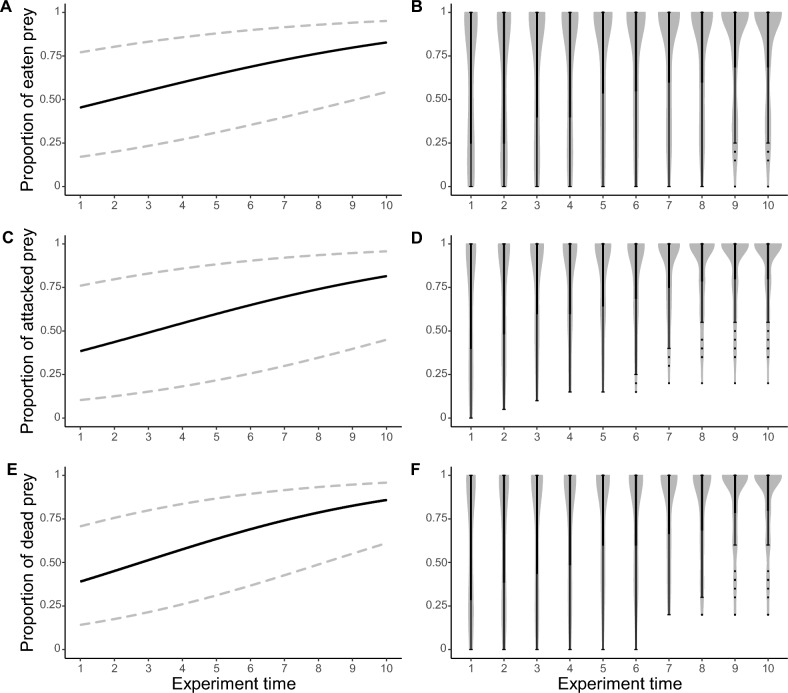
Trends for the best generalized linear mixed models testing for the drivers of the proportion of eaten, attacked and dead prey as a function of the duration of the experiment (Experiment time, **A**, **C**, **E**, respectively). Lower and upper 95 % confidence intervals are represented by the dashed lines. Violin boxplots for the proportion of eaten, attacked or dead prey are a combination of kernel density plots and boxplots. For each experiment time (n = 72 replicates), violin boxplots represent the distribution of the raw data, and the upper adjacent value, third quartile, median, first quartile and lower adjacent value from top to bottom (**B**, **D**, **F**, respectively).

**Figure 3:**
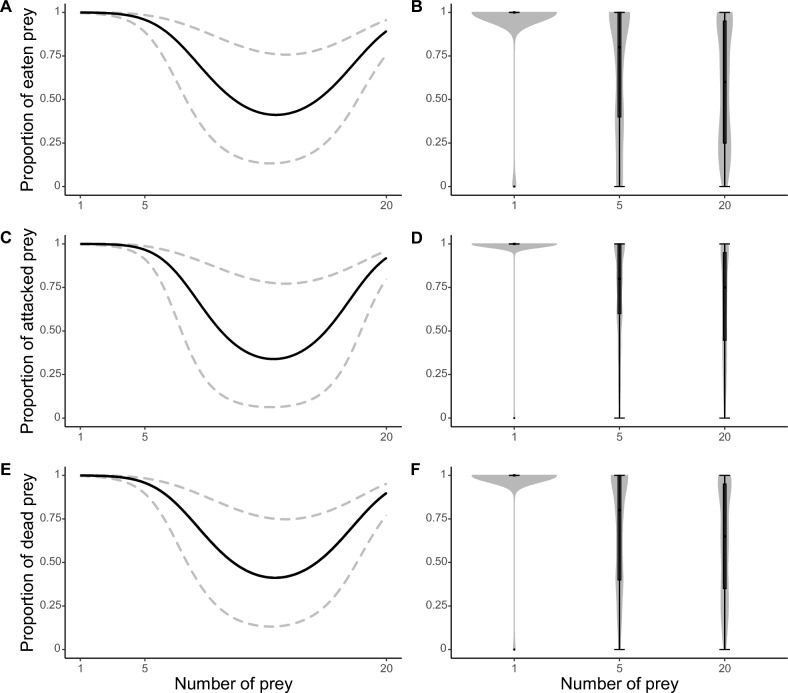
Trends for the best generalized linear mixed models testing for the drivers of the proportion of eaten, attacked and dead prey as a function of the number of prey (**A**, **C**, **E**, respectively). Lower and upper 95 % confidence intervals are represented by the dashed lines. Violin boxplots for the proportion of eaten, attacked or dead prey are a combination of kernel density plots and boxplots. For each prey density (n = 240 replicates), violin boxplots represent the distribution of the raw data, and the upper adjacent value, third quartile, median, first quartile and lower adjacent value from top to bottom (**B**, **D**, **F**, respectively).

The proportion of eaten, attacked and dead larvae of the fly *Fucellia maritima* (Haliday 1838) (Diptera: Anthomyiidae) varied in a similar way in response to the numbers of predators, the quadratic term of the predator number variable, and the duration of the experiment (Table 1). Conversely, an opposite relationship was found for the number of prey and the quadratic term of the number of prey variable (Table 1). Specifically, the number of eaten, attacked or dead prey increased with increasing numbers of *M. soledadinus*, and with increasing duration of the experiment, while the number of eaten, attacked or dead prey decreased with increasing prey density (Table 1, Figs. 1, 2 and 3). The proportions of eaten, attacked and dead prey were strongly correlated (Pearson coefficient of: 0.90 for the correlation between the proportion of eaten and attacked prey, − 0.94 for the correlation between the proportion of eaten and dead prey, − 0.93 for the correlation between the proportion of attacked and dead prey).

**Table 1:** Estimates, standard errors (between brackets) and model parameters for the best generalized linear mixed models testing for the drivers of the proportion of eaten, attacked and dead prey due to *M. soledadinus* (see “Methods” for more details about the statistical analyses).

	Proportion of eaten prey	Proportion of attacked prey	Proportion of dead prey
(Intercept)	3.37*** (0.92)	5.69*** (1.52)	3.90*** (0.99)
Experiment time	0.19*** (0.01)	0.22*** (0.01)	0.25*** (0.01)
Predator number	0.30*** (0.05)	0.21*** (0.05)	0.24*** (0.05)
Predator number^2^	< − 0.01*** (< 0.01)	< − 0.01** (< 0.01)	< − 0.01** (< 0.01)
Number of prey	− 1.38*** (0.27)	− 1.64*** (0.40)	− 1.39*** (0.27)
Number of prey^2^	0.05*** (0.01)	0.06*** (0.02)	0.05*** (0.01)

Akaike’s Information Criteria (AIC)	1233.50	1227.44	1257.76
Bayesian Information Criteria (BIC)	1265.56	1259.50	1289.82
Log likelihood	− 609.75	− 606.72	− 621.88
Number of observations	720	720	720
Number of groups: ID “Experimental box”	72	72	72
Variance: ID “Experimental box” (Intercept)	2.34	1.97	1.91

Conditional R^2^	0.97	0.97	0.97
Marginal R^2^	0.78	0.81	0.80

The conditional pseudo-R^2^ corresponds to the variance explained by the entire model, whereas the marginal pseudo-R^2^ corresponds to the variance explained by the fixed effects only.

Significant effects are coded as follows: ***p < 0.001; **p < 0.01; *p < 0.05.

The proportion of eaten, attacked or dead prey significantly increased at predator densities of more than five individuals, and then stabilized at maximum values when 20 and 50 predators were present (Fig. 1). The mean proportions and the variance of eaten prey for 1, 5, 20 and 50 predators were 0.45, 0.67, 0.88 and 0.96; and 1, 0.95, 0.75 and 0.40, respectively (see Supplementary material 3, Table 1 for the mean proportion and variance of attacked and dead prey as a function of the number of predators, calculated at the end of the experiment).

The proportion of eaten, attacked or dead prey steadily rose as experimental duration increased, reaching a maximum at the end of the experiment (experiment time point 10; Fig. 2). The mean proportion and the variance of eaten prey for experiment time points 1 and 10 reached 0.63 and 0.80, and 1 and 1, respectively (see Supplementary material 3, Table 2 for the mean proportion and variance of attacked and dead prey in function of the experiment time point, calculated at the end of the experiment).

Conversely, while a sharp decline occurred when the prey density was at five, the proportion of eaten, attacked or dead prey continued to decrease as prey density increased (20 prey, Fig. 3A, C and E). Specifically, the mean proportion of eaten prey when the density prey was at 1, 5 and 20 prey equaled 0.97, 0.67 and 0.58 respectively; with a variance of 1 for all prey densities (see Supplementary material 3, Table 3 for the mean proportion and variance of attacked and dead prey as a function of the number of prey, calculated at the end of the experiment).

The residence time, body mass and the sex ratio of *M. soledadinus* were not retained in the best models, and were not significant in the full models (Table 1, Supplementary material 4, Table 1). These variables did not significantly explain the variation in the proportion of eaten, attacked or dead prey by *M. soledadinus*.

Finally, the impact potential values varied considerably between the populations, but did not vary as a function of their residence time. Impact potential exhibited a ratio of 7.5 between the lowest and highest values, i.e. ranging from 196.5 to 1500, with a mean of 663 (Table 2). The highest impacts were projected for the populations at Pointe Suzanne (1500) and Ratmanoff (800) which are two of the populations with the shortest residence times (10 and 8 years, respectively). Populations of *M. soledadinus* with the longest residence times did not have the highest abundances, and those with the shortest residence times did not have the highest maximum feeding rates (Table 2).

**Table 2:** Information on residence time, maximum feeding rate, mean abundance and potential impact for each population.

Population	Residence time (years)	Maximum feeding rate	Mean abundance	Impact potential
Isthme Bas	5	5	100	500
Ratmanoff	8	8	100	800
Pointe Suzanne	10	15	100	1500
Anse des Pachas	21	3	65.5	196.5
Cataractes	36	10	65.5	655
Port Elizabeth	46	5	65.5	327.5

Impact potential was calculated as the product of maximum feeding rate and abundance, following Dick et al.^52^.

## Discussion

The impacts of the non-native invasive beetle *M. soledadinus* appeared to be independent of residence time, body mass and sex in our study, and therefore their effects on resident fauna may be widespread. The number of adult *M. soledadinus* and the duration of the experiment had a significant positive influence on the proportion of eaten, attacked and dead prey. The number of prey significantly negatively influenced the proportion of eaten, attacked and dead prey. Conversely, the residence time of the population, sex ratio and body mass were not significant predictors of the strength of predator–prey interaction. Moreover, population-level impacts were not formally correlated with residence time even if some of the populations with the shortest residence times had high impact potential.

*Per capita* feeding rates of invasive species can be linked to their ecological effects, and the impact potential metric applied here has been found to correlate with independent assessments of ecological impacts in the field^52^. The proportion of eaten, attacked and dead prey rapidly increased before plateauing at higher densities of *M. soledadinus*. However, this “saturation” in the effect of the predator density (visible by the significant negative quadratic term of the number of predators) might also partly result from progressive prey depletion. Prey were not replaced during the experiment, which represents a possible experimental bias^53^. The proportion of eaten, attacked and dead prey first decreased at the intermediate prey density but then increased towards the highest prey density, potentially revealing a form of “predator learning”, or an increased efficiency of foraging as the number of prey increased. Learning in foraging and prey selection have been identified in insects^54–56^. Further examination of this theory should include additional experimental treatments in order to cover a higher range of predator–prey densities.

Residence time can influence a myriad of physiological and morphological traits in invasive species. Former investigations found that the dispersal capacities of adult *M. soledadinus*, and their morphology, varied as a function of their residence time: heavier and larger individuals were collected in the populations having the shortest residence times in comparison with the populations having the longest residence times (3 and 11 years, 36 and 93 years, respectively)^57^. Since we did not find any clear residence time effect, in situ field studies and inclusion of other sites in future that have a more longstanding invasion would be beneficial. Interestingly, body mass, sex or residence time of populations of *M. soledadinus* had no effect on the proportion of eaten, attacked or dead prey in our study. This finding suggests a lack of effect of these context-dependencies in the predator–prey relationship among the different populations of *M. soledadinus* regarding the prey used in this study. By maintaining a constantly high predation rate, the impacts caused by this species of beetle on native insect communities could therefore be immediate and severe, albeit non-linear, escalating with its increasing density, especially where prey are limited.

Our study provides a new insight into predation dynamics, as we uniquely studied the predator–prey relationships of *M. soledadinus* with three response variables: the proportion of eaten, attacked or dead prey. While the energy consumption of *M. soledadinus* was visible via the proportion of eaten prey, the proportion of attacked prey gave an indication on predation, and the possible existence of wasteful killing. The proportion of dead prey provided information on a combination of the effects of *M. soledadinus* on prey survival and on ‘natural’ prey death. It is important to account for potential wasteful killing by predators, as this phenomenon has been often noted in insects^58–61^. Moreover, wasteful killing behavior in predators can have very important negative consequences for the density of prey populations^62^. For *M. soledadinus*, we found no evidence of wasteful killing; the same decreasing trend for the proportion of eaten, attacked and dead prey was measured even as prey densities increased. Yet, conclusions made from the present results must also be considered with caution, as they might vary with the type of prey used, and are not completely reflective of natural conditions^53^.

Our results using the non-native prey species *F. maritima* suggest that *M. soledadinus* is likely to have a much higher impact on the insect fauna, native and non-native, in areas where its population densities are very high, in line with former investigations on other non-native predators invading different environments^63^. Indeed, we measured an increased proportion of eaten, attacked and dead prey with increasing densities of this beetle species. On the Kerguelen Islands, *M. soledadinus* can be found in high densities in some areas, with up to 100 individuals found during a ten-minute count a single location^48^. Such densities clearly exceed the maximum predator density we applied in our experiment. Moreover, current densities recorded of *M. soledadinus* are considerably higher than those reported in the 1990s^48^, which would suggest that polar amplification boosts population dynamics and increases the impacts of this predatory non-native insect on native communities.

Habitat complexity is an important consideration when measuring predator–prey dynamics. The addition of a sand layer at the bottom of the experimental boxes is not only useful to maintain a suitable humidity for *M. soledadinus* which only survives in moist conditions, but also to allow *F. maritima* to be somewhat inconspicuous, so as not to influence predation pressure^64^. The sand substrate also acts as a potential refuge for *F. maritima.* Refuge effects have been widely recognized as prevailing in predator–prey relationships, with a higher concentration of refuges leading to reduced predator impacts due to an increased searching time for prey^65,66^. However, even if the relatively shallow sandy substrate we used was not entirely comparable to field conditions, it was the most realistic experimental design that could be used, as both *M. soledadinus* and *F. maritima* are found in coastal environments on Kerguelen Islands^53,67^.

Predation rates were prey density-dependent in the present study. We found that the prey consumption rate by *M. soledadinus* decreased with increasing prey density (at least with its linear term), despite the restricted prey densities we studied in our experiment. This pattern could correspond to the most common type-two functional response, whereby prey consumption rates fall as their density increases (thus following a hyperbolic form)^32^. However, experiments using a broader range of prey densities than assessed here would be required to test this theory. Our generalized conclusions on the impacts of *M. soledadinus* across the native invertebrate community on the island are restricted by our experiments using a single non-native prey species, and therefore patterns might be different when focusing on native prey species. Meanwhile, it is important to consider the predator–prey dynamics between *F. maritima* and *M. soledadinus*, as the non-native *F. maritima* is present in high densities in coastal areas of the Kerguelen Islands and its larvae represent an important part of the beetle’s diet^27,67^. Therefore, exploring greater prey and predator densities, or ideally including native prey species, despite their vulnerability, would allow us to expand our knowledge of the impacts of *M. soledadinus* in its invaded range on the Kerguelen Islands.

Contrary to our expectations, and to previous findings on other predator species^35,36,49^, the residence time of the populations, the sex or the body mass of *M. soledadinus* did not affect its predation rate. We found that the impact of *M. soledadinus* was relatively high no matter its residence time. Indeed, predator density was the most important characteristic explaining the impact of *M. soledadinus* overruling its other characteristics such as residence time, sex and body mass. Additionally, even if populations did not appear to have significantly increasing impact values in relation to decreasing residence times compared to some other non-native predators^49^, we found that some populations with short residence times had very high potential impact when using *F. maritima* as the model prey species. Thus, a pattern of increased impact along the invasion gradient tended to appear for these populations that combined short residence times and high potential impacts, in line with the spatial sorting concept which is often associated with increased performance of invasive organisms at the invasion front^67,68^. The relatively low observed potential impact of the population of *M. soledadinus* from Isthme Bas may be a consequence of a much faster increase in population density than in other localities. Indeed similar prey capture rates to those of a long-established population were found at Isthme Bas, *ca.* 3 years after the arrival of *M. soledadinus*^48^. Such prey-capture rates may have quickly increased intraspecific competition and altered the trophic behaviour of *M. soledadinus*. Generally, the present results would imply that *M. soledadinus* might have important and rapid impacts on the native insect communities, in line with the observed rapid decline of the native—and sometimes endemic—fauna of the Kerguelen Islands^11,27,46^. Indeed, in the presence of high numbers of *M. soledadinus* on the Kerguelen Islands, the densities of native arthropods and especially of native flightless insects—which are inherently naive to ground-based predators—are negatively impacted.

For example, two flightless native species display important negative impact from the presence of *M. soledadinus* on Kerguelen Islands. Populations of the native *Anatalanta aptera* (Eaton 1875, Diptera: Sphaeroceridae) are highly reduced, while the native *Calycopteryx moseleyi* (Eaton 1875, Diptera: Micropezidae) has been locally driven to extinction^48^. On Kerguelen Islands, it has to be noted that there may be competition between native prey species of *M. soledadinus* such as *A. aptera* or *C. moseleyi*, and non-native prey species of *M. soledadinus* such as *F. maritima*, which are not taken into account in our study. These competition impacts could play an important role in the decline of populations of native prey species in addition to the negative influence of predation by *M. soledadinus*. However, prey species such as *C moseleyi* and *F. maritima* strongly respond to increases in food availability. For example, *C. moseleyi* preferably feeds on decomposing tissues of the native plant species *Pringlea antiscorbutica*, while *F. maritima* is mainly found in seaweed. In areas where multiple food sources are found, those two species seem to have little impact on each other via competition^69^. For now, the sharp declines in native prey species on Kerguelen Islands are mainly attributed to the predation by *M. soledadinus*, although further studies on competition between native and non-native prey species should be done to elucidate how multiple impact mechanisms might combine, as well as the effects of this invader on native prey species^48^. Finally, some places on the Kerguelen Islands are not yet invaded by *M. soledadinus*, due to barrier zones such as cliffs or penguin colonies. At these sites, native insect species remain unimpacted by this non-native beetle^48^.

The distribution and survival of many native species from subantarctic islands are highly influenced by global changes, notably by the intense temperature warming and precipitation changes occurring in the region^70–72^. Long-term monitoring of *M. soledadinus* on Kerguelen Islands provides valuable insights into its distribution and range expansion since the 1990’s, and the colonization of the beetle towards higher elevations^48^. Native species are already migrating to higher elevations on subantarctic islands^73^. However, on Kerguelen Islands, such altitudinal expansion is likely to be also under pressure from the negative impacts of *M. soledadinus*, since this non-native predatory species progresses rapidly in areas with abundant food resources^64^. Additionally, *M. soledadinus* displays limited stress signals in warming conditions (as high as 20 °C), which would indicate that temperature warming on Kerguelen Islands might not limit individuals in their further spread, but could even stimulate them, for example by intensifying predation behavior^14,48,74^. However, the decreased precipitation induced by climate change on Kerguelen Islands could be problematic for *M. soledadinus*, if the areas where it is present become drier, as has been noted for some wetlands and ponds with the decline of native wetland plant species^10,11,75,76^. Indeed, it has been found in experimental settings that the survival of this beetle is reduced in drier conditions^64^.

*M. soledadinus* is a non-native predator species that already seems to have very deleterious impacts on the native fauna on subantarctic islands where it has invaded^11,27^. Such impacts are at risk of exacerbation in the future, as climatic changes within the region further weaken and stress native biota, and also possibly bolster the metabolism of this non-native insect predator^11,20,22^. Furthermore, this non-native predator species might entirely disrupt ecosystem functioning. Indeed, the native species that *M. soledadinus* appears to threaten play a significant role in decomposition activities^12,13^. Once *M. soledadinus* reached the scientific research station on the Kerguelen Islands, the only part of the archipelago with sustained anthropogenic influence, its spread rapidly increased, assisting its reach to isolated locations^48^. It is therefore essential to mitigate the risks of further anthropogenic assistance in the continued spread of *M. soledadinus*. The declaration of the Kerguelen Islands as a Nature Reserve in 2006 has resulted in the development and application of biosecurity protocols that may assist managers to control the further spread of *M. soledadinus*, and limit the risk of future non-native species introductions^77,78^. In the future, it will be important to limit the importation of individuals of *M. soledadinus* to areas on the Kerguelen Islands that are still free of this species, but also to ensure it does not invade other cold environments.

## Methods

### Insect collection

**Figure 4:**
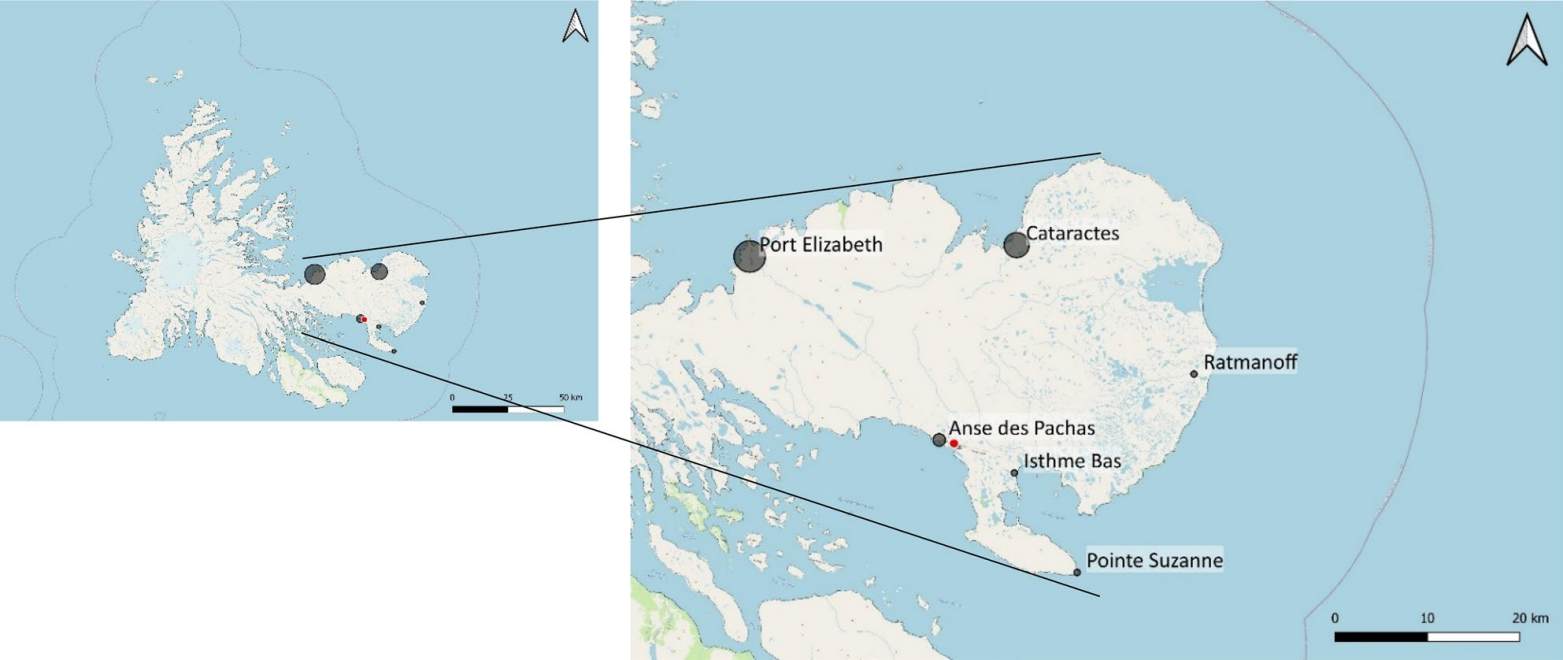
Locations of the populations of *M. soledadinus* on the Kerguelen Islands are represented by grey dots, with increasing dot size corresponding to increasing residence time: 46 years at Port Elizabeth, 36 years at Cataractes, 21 years at Anse du Pacha, 10 years at Pointe Suzanne, eight years at Ratmanoff and five years at Isthme Bas. The red dot corresponds to the research station of Port-aux-Français. The map of the Kerguelen Islands was created by the authors. The background map comes from ©OpenStreetMap^79^; OpenStreetMap is *open data* (Open Data Commons Open Database License, ODbL, and license CC BY-SA 2.0 for the documentation). QGIS 3.28.8 LTR was used to generate the map (www.qgis.org).

In 2015, 350 live adult *M. soledadinus* were hand-collected from each of the six following localities of the Kerguelen Islands: Port Elizabeth, Cataractes, Anse du Pacha, Pointe Suzanne, Ratmanoff and Isthme Bas (Fig. 4). All of these localities are coastal, and host large densities of *M. soledadinus* with known residence times. At the time of collection, *M. soledadinus* was established for 46 years at Port Elizabeth, 36 years at Cataractes, 21 years at Anse du Pacha, 10 years at Pointe Suzanne, eight years at Ratmanoff and five years at Isthme Bas (Fig. 4) (Source: project 136—SUBANTECO, French polar institute). Adults from these six localities were deemed to belong to different populations. Based on the fact that the average adult longevity of *M. soledadinus* is about eight months^75^ and may produce two generations per year, the number of generations since the establishment of the insect in each locality would vary at least from seven (for the population with the shortest residence time used in our study: Isthme Bas) to 92 generations (for the population with the longest residence time used in our study: Port Elizabeth).

### Insect maintenance

All collected individuals were transported live the day after the collection, in sealed plastic boxes, to the research station laboratory (Port-aux-Français, Kerguelen Islands). Then, each population was immediately placed in separate plastic boxes (18 × 12 × 7 cm) at standardized densities of 150 individuals per box (total of 350 individuals for each population, distributed in two boxes with 150 individuals each, and a back-up box with 50 individuals). Sand collected at the Anse du Pacha locality (49°21′01.3"S, 70°11′52.6"E) was thoroughly rinsed with tap water, drained and left moist, and a 5 mm layer was placed at the bottom of each box before the transfer of the insects. Adults of *M. soledadinus* cannot survive in low air humidity conditions (relative humidity < 70%), or if they lack access to fresh water^64^. Hence, this sandy substrate provided adults with their humidity and water requirements. Rinsed wood pieces or gravel were added to provide adults with shelters. The plastic boxes containing the insects collected from the different populations were then placed in a walk-in climatic chamber at 8 °C, photoperiod 12:12 (Light:Dark).

Plastic boxes of all populations were checked once per week to control the stability of air and soil humidity. To that aim, they were opened 1 h per week for the renewal of the air, and the humidity of the substrate was readjusted with tap water to keep it appropriately moist. During the weekly checks, larvae of *M. soledadinus* were removed, if present. At the same time, adults of *M. soledadinus* were fed weekly with larvae of the fly *F. maritima*. This dipteran is a non-native species present in high densities in coastal environments on the Kerguelen Islands, and is a large part of the diet of *M. soledadinus* there^27,75,80^. Furthermore, *F. maritima* is very similar in size, habitat and diet during larval stages to *A. aptera* or *C. moseleyi*, two example native fly species also consumed by *M. soledadinus*^67^. So far as we are aware, these other fly species are naive to insect predators, and the use of *F. maritima* as a prey had less impact on insect communities than if we had collected a native species. One week prior to the beginning of the experiment, feeding of all *M. soledadinus* was stopped, so that the beetles used for the experiments were likely to have cleared guts^67^.

### Experimental design and setup

Transparent plastic boxes (18 × 12 × 7 cm) were filled with 2 mm of tap water-rinsed sand from the aforementioned location of Anse du Pacha. The shallower depth of sand in the boxes was applied to avoid larval digging, thereby reducing their ability to hide from *M. soledadinus*. One plastic box was assigned to a single treatment combination: population of the predator (i.e., *M. soledadinus*: Port Elizabeth, Cataractes, Anse du Pacha, Pointe Suzanne, Ratmanoff and Isthme Bas) × number of predators (1, 5, 20, or 50 individuals) x number of prey (i.e., *F. maritima*: 1, 5, or 20 individuals), resulting in a total of 72 treatment combinations. We had one experimental replicate per treatment combination. As we needed 76 M*. soledadinus* individuals for each population for the predator treatment, we used the individuals collected from the two plastic boxes of 150 individuals (Insects from the box containing 50 individuals were not used). At the start of each experiment, plastic boxes were placed in a single walk-in climatic chamber at 8 °C, photoperiod 12:12 (L:D), and the designated number of predators from one out of the six studied populations of *M. soledadinus* was paired with the designated number of prey. The experiment lasted for five days, and began at 18 h on day 0. We defined the experiment time point as the different time points during which the prey-predator interactions were observed over the course of the experiment, ranging from observations at experiment time point 1 to 10. Thereby, the condition of the prey (i.e., analyzed response variables) was checked two times a day with a binocular magnifier installed in the walk-in climatic chamber, at 9.00 am (experiment time point 1) and 6.00 pm (experiment time point 2) at day 1, and repeated every day until 6.00 pm on day 5 (experiment time point 10) (Supplementary material 1, Table 1). At each experiment time point, prey were classified as dead or alive, attacked or non-attacked, eaten or non-eaten. At the end of the assay, *M. soledadinus* individuals were killed, weighed (body mass in mg), and their sex was determined.

### Statistical analyses

Based on the classification of the prey, we calculated: the number of eaten, non-eaten, attacked, non-attacked, dead and alive prey. To do so, the number of eaten prey was calculated as: $partially\, eaten\, larvae\, +\, entirely \,eaten\, larvae$; while the number of non-eaten prey was calculated as: $alive\, larvae\, +\, dead\, larvae\, +\, alive \,wounded\, larvae\, +\, dead\, wounded \,larvae$. The number of attacked prey was calculated as: $alive \,wounded\, larvae\, + \,dead\, wounded\, larvae\, +\, partially\, eaten\, larvae\, +\, entirely\, eaten\, larvae$; the number of non-attacked prey was calculated as: $alive\, larvae\, + \,dead\, larvae$. The number of dead prey was calculated as: $dead\, larvae \,+\, dead\, wounded\, larvae\, +\, partially\, eaten\, larvae\, +\, entirely \,eaten\, larvae$; while the number of alive prey was calculated as: $alive\, larvae\, + \,alive\, wounded \,larvae$. The number of eaten or non-eaten prey informed the energy consumption of *M. soledadinus*. The number of attacked and non-attacked prey indicated the ecological impact of *M. soledadinus* on *F. maritima* including wasteful killing. The number of dead and alive prey gave an indication on the influence of predation, but also on death of larvae of *F. maritima* not related to predator attacks. Since we were interested on the effects of different numbers of *M. soledadinus* on *F. maritima*, we took into account their sex ratio and their mean body mass for each treatment combination. We thus calculated the ratio of female *M. soledadinus* as: $\frac{number \,of \,female\, M. soledadinus}{total\, number\, of \,M. soledadinus}\times 100$, and the mean body mass of *M. soledadinus* individuals.

We analyzed three response variables: the proportion of eaten prey, the proportion of attacked prey and the proportion of dead prey. These response variables allowed us to focus on slightly different signals depicting the effects of the predator *M. soledadinus* on the prey *F. maritima*. To do so, we used a binomial denominator written as a vector: number of successes, number of failures^81^, corresponding in the present work to the pairs: number of eaten prey, number of non-eaten prey; number of attacked prey, number of non-attacked prey; and number of dead prey, number of alive prey, respectively. The relationships between the response variables (proportion of eaten prey, proportion of attacked prey, proportion of dead prey) and the explanatory variables (number of prey (continuous) and number of predators (continuous), mean body mass (continuous), residence time (continuous), ratio of female predators (continuous), and duration of the experiment (continuous)) were analyzed using generalized linear mixed models with a binomial family and a logit link (See Supplementary Material 2 for model structure, R package lme4, Ref.^82^). We included the quadratic terms for the number of prey and the number of predators. We also included the second order interactions between: the number of predators and the residence time, the residence time and the female ratio, the residence time and the mean body mass. The addition of the identity of each independent plastic box as a random intercept was necessary to account for the experimental design, and increased the fit of the mixed models for each of the response variables (Akaike Information Criterion (AIC) decreased with more than two units, Ref.^83^. The *dredge* function on the full models was used for each response variable (package MuMIn, Ref.^84^) to select the best model (the one with the lowest AIC and with the highest Phi coefficient (model precision)). We did not detect zero inflation for the best models for each of the response variable (DHARMa residuals diagnostic, R package DHARMa, Ref.^85^). For each best model, the pseudo-R-squared was calculated (R package MuMIn, Ref.^84^).

Following Dick et al.^52^, an adaptation of the “impact potential” metric was employed, and calculated as: $abundance\, \times \,maximum\, feeding\, rate$. The rounded abundance was estimated in the field as a mean number of *M. soledadinus* adults at each of the population locations sampled. The maximum feeding rate was determined from the number of eaten prey at the last experiment time point for each population, from each single treatment condition: the lowest predator density (one predator) and the highest prey density (20 prey). This allowed the *per capita* effect and the population-level effect to be combined and compared, to gain a fuller understanding of ecological impact among beetle populations.

All statistical analyses were performed in R, version 3.5.2^86^ and *p* = 0.05 was taken as threshold for significance.

### Supplementary Information

Supplementary Information.

## Data Availability

The data presented in this study are openly available in GitHub at https://github.com/davidrenault/Merizodus_predator_prey.

## Code availability

R scripts from the analyses in this article were developed by CC with the help of RNC and are openly available in GitHub at https://github.com/davidrenault/Merizodus_predator_prey.
