# Supplementary material for: Density-dependent predatory impacts of an invasive beetle across a subantarctic archipelago
Source: Sci Rep. 2023 Sep 2;13:14456. doi: 10.1038/s41598-023-41089-2 (PMC10475102; doi:10.1038/s41598-023-41089-2)
Supplement: Supplementary file 1 — Supplementary Information. [file 41598_2023_41089_MOESM1_ESM.docx]

**Supplementary material 1**

Table 1: details of the experiment times and the corresponding days and hours during the experiment.

| Experiment time | Day | Hour |
| --- | --- | --- |
| 1 | 1 | 9h00 |
| 2 | 1 | 18h00 |
| 3 | 2 | 9h00 |
| 4 | 2 | 18h00 |
| 5 | 3 | 9h00 |
| 6 | 3 | 18h00 |
| 7 | 4 | 9h00 |
| 8 | 4 | 18h00 |
| 9 | 5 | 9h00 |
| 10 | 5 | 18h00 |

**Supplementary material 2**

Structures of the full and best models for each response variable:

*Full models*

ProportionOfEatenPrey <- glmer(cbind(NumberOfEatenPrey, NumberOfNon-EatenPrey)~ ((ResidenceTime + NumberOfPrey + NumberOfPrey² + NumberOfPredators + NumberOfPredators² + ExperimentTime + MeanBodyMass + PercentageOfFemales + NumberOfPredators*ResidenceTime + ResidenceTime*PercentageOfFemales + ResidenceTime*MeanBodyMass)) + (1|PlasticBoxIdentity), family = binomial(link="logit"))

ProportionOfAttackedPrey <- glmer(cbind(NumberOfAttackedPrey, NumberOfNon-AttackedPrey)~ ((ResidenceTime + NumberOfPrey + NumberOfPrey² + NumberOfPredators + NumberOfPredators² + ExperimentTime + MeanBodyMass + PercentageOfFemales + NumberOfPredators*ResidenceTime + ResidenceTime*PercentageOfFemales + ResidenceTime*MeanBodyMass)) + (1|PlasticBoxIdentity), family = binomial(link="logit"))

ProportionOfDeadPrey <- glmer(cbind(NumberOfDeadPrey, NumberOfAlivePrey)~ ((ResidenceTime + NumberOfPrey + NumberOfPrey² + NumberOfPredators + NumberOfPredators² + ExperimentTime + MeanBodyMass + PercentageOfFemales + NumberOfPredators*ResidenceTime + ResidenceTime*PercentageOfFemales + ResidenceTime*MeanBodyMass)) + (1|PlasticBoxIdentity), family = binomial(link="logit"))

*Best models*

ProportionOfEatenPrey <- glmer(cbind(NumberOfEatenPrey, NumberOfNon-EatenPrey)~ ((NumberOfPrey + NumberOfPrey² + NumberOfPredators + NumberOfPredators² + ExperimentTime)) + (1|PlasticBoxIdentity), family = binomial(link="logit"))

ProportionOfAttackedPrey <- glmer(cbind(NumberOfAttackedPrey, NumberOfNon-AttackedPrey)~ ((NumberOfPrey + NumberOfPrey² + NumberOfPredators + NumberOfPredators² + ExperimentTime)) + (1|PlasticBoxIdentity), family = binomial(link="logit"))

ProportionOfDeadPrey <- glmer(cbind(NumberOfDeadPrey, NumberOfAlivePrey)~ ((NumberOfPrey + NumberOfPrey² + NumberOfPredators + NumberOfPredators² + ExperimentTime)) + (1|PlasticBoxIdentity), family = binomial(link="logit"))

**Supplementary material 3**

Table 1: Mean, minimum and maximum values of the proportion (“Prop.”) of eaten, attacked and dead prey depending on the number of predators, calculated at the end of the experiment.

| Number of predators | Prop. eaten mean | Prop. eaten min | Prop. eaten max | Prop. attacked mean | Prop. attacked min | Prop. attacked max | Prop. dead mean | Prop. dead min | Prop. dead max |
| --- | --- | --- | --- | --- | --- | --- | --- | --- | --- |
| 1 | 0.449 | 0.00 | 1 | 0.641 | 0.00 | 1 | 0.557 | 0.00 | 1 |
| 5 | 0.669 | 0.05 | 1 | 0.761 | 0.15 | 1 | 0.710 | 0.10 | 1 |
| 20 | 0.878 | 0.25 | 1 | 0.899 | 0.30 | 1 | 0.887 | 0.25 | 1 |
| 50 | 0.961 | 0.60 | 1 | 0.967 | 0.70 | 1 | 0.962 | 0.60 | 1 |

Table 2: Mean, minimum and maximum values of the proportion (“Prop”) of eaten, attacked and dead prey depending on the experiment time, calculated at the end of the experiment.

| Experiment time | Prop. eaten mean | Prop. eaten min | Prop. eaten max | Prop. attacked mean | Prop. attacked min | Prop. attacked max | Prop. dead mean | Prop. dead min | Prop. dead max |
| --- | --- | --- | --- | --- | --- | --- | --- | --- | --- |
| 1 | 0.627 | 0 | 1 | 0.701 | 0.00 | 1 | 0.656 | 0 | 1 |
| 2 | 0.652 | 0 | 1 | 0.752 | 0.05 | 1 | 0.687 | 0 | 1 |
| 3 | 0.709 | 0 | 1 | 0.789 | 0.10 | 1 | 0.740 | 0 | 1 |
| 4 | 0.717 | 0 | 1 | 0.802 | 0.15 | 1 | 0.755 | 0 | 1 |
| 5 | 0.754 | 0 | 1 | 0.827 | 0.15 | 1 | 0.783 | 0 | 1 |
| 6 | 0.760 | 0 | 1 | 0.835 | 0.15 | 1 | 0.790 | 0 | 1 |
| 7 | 0.784 | 0 | 1 | 0.859 | 0.20 | 1 | 0.828 | 0.2 | 1 |
| 8 | 0.787 | 0 | 1 | 0.861 | 0.20 | 1 | 0.838 | 0.2 | 1 |
| 9 | 0.801 | 0 | 1 | 0.871 | 0.20 | 1 | 0.856 | 0.2 | 1 |
| 10 | 0.803 | 0 | 1 | 0.872 | 0.20 | 1 | 0.860 | 0.2 | 1 |

Table 3: Mean, minimum and maximum values of the proportion (“Prop”) of eaten, attacked and dead prey depending on the number of prey, calculated at the end of the experiment.

| Number of prey | Prop. eaten mean | Prop. eaten min | Prop. eaten max | Prop. attacked mean | Prop. attacked min | Prop. attacked max | Prop. dead mean | Prop. dead min | Prop. dead max |
| --- | --- | --- | --- | --- | --- | --- | --- | --- | --- |
| 1 | 0.967 | 0 | 1 | 0.996 | 0 | 1 | 0.983 | 0 | 1 |
| 5 | 0.668 | 0 | 1 | 0.775 | 0 | 1 | 0.719 | 0 | 1 |
| 20 | 0.584 | 0 | 1 | 0.680 | 0 | 1 | 0.635 | 0 | 1 |

**Supplementary material 4**

Table 1: Estimates, standard errors (between brackets) and model parameters for the full models testing for the drivers of the proportion of eaten, attacked and dead prey due to *M. soledadinus*. Significant effects are coded as follows: ***p < 0.001; **p < 0.01; *p < 0.05.

|  | **Proportion of eaten prey** | **Proportion of attacked prey** | **Proportion of dead prey** |  |  |
| --- | --- | --- | --- | --- | --- |
| (Intercept) | 0.92 (4.25) | 2.75 (4.53) | 0.85 (4.03) |  |  |
| Residence time | 0.34 (0.24) | 0.29 (0.25) | 0.37 (0.24) |  |  |
| Prey number | -1.26^***^ (0.26) | -1.58^***^ (0.40) | -1.32^***^ (0.28) |  |  |
| Prey number² | 0.05^***^ (0.01) | 0.06^***^ (0.02) | 0.05^***^ (0.01) |  |  |
| Predator number | 0.27^***^ (0.06) | 0.20^***^ (0.06) | 0.22^***^ (0.06) |  |  |
| Predator number² | <-0.01^**^ (<0.01) | <-0.01^*^ (<0.01) | <-0.01^**^ (<0.01) |  |  |
| Experiment time | 0.19^***^ (0.01) | 0.22^***^ (0.01) | 0.25^***^ (0.01) |  |  |
| Mean body mass (mg) | 0.27 (0.48) | 0.33 (0.49) | 0.38 (0.46) |  |  |
| Female to male ratio (%) | <0.01 (0.02) | <0.01 (0.02) | -0.01 (0.02) |  |  |
| Residence time × Predator number | <-0.01 (<0.01) | <-0.01 (<0.01) | <-0.01 (<0.01) |  |  |
| Residence time × Female to male ratio | <0.01 (<0.01) | <0.01 (<0.01) | <0.01 (<0.01) |  |  |
| Residence time × Mean body mass | -0.04 (0.03) | -0.04 (0.03) | -0.05 (0.03) |  |  |
| AIC | 1241.12 | 1237.05 | 1265.30 |  |  |
| BIC | 1300.65 | 1296.58 | 1324.83 |  |  |
| Log Likelihood | -607.56 | -605.53 | -619.65 |  |  |
| Num. obs. | 720 | 720 | 720 |  |  |
| Num. groups: ID “Experimental box” | 72 | 72 | 72 |  |  |
| Var: ID “Experimental box” (Intercept) | 2.07 | 1.87 | 1.71 |  |  |
|  | | | | |  |
